# Factors and Prognostic Significance of Impaired Exercise Tolerance in Women over 40 with Arterial Hypertension

**DOI:** 10.3390/jpm11080759

**Published:** 2021-07-30

**Authors:** Agata Bielecka-Dabrowa, Katarzyna Gryglewska, Agata Sakowicz, Stephan von Haehling, Kamil Janikowski, Marek Maciejewski, Maciej Banach

**Affiliations:** 1 Heart Failure Unit, Department of Cardiology and Congenital Diseases of Adults, Polish Mother’s Memorial Hospital Research Institute (PMMHRI), 93-338 Lodz, Poland; kkjanikowski@gmail.com (K.J.); maciejbanach77@gmail.com (M.B.); 2 Department of Preventive Cardiology and Lipidology, Chair of Nephrology and Hypertension, Medical University of Lodz, 93-338 Lodz, Poland; 3 Department of Cardiology and Congenital Diseases of Adults, Polish Mother’s Memorial Hospital Research Institute (PMMHRI), 93-338 Lodz, Poland; marek.maciejewski@iczmp.edu.pl; 4 Department of Medical Biotechnology, Medical University of Lodz, 93-338 Lodz, Poland; agata.sakowicz@gmail.com; 5 Department of Cardiology and Pneumology and German Center for Cardiovascular Research (DZHK), Partner Site Göttingen, University Medical Center Göttingen (UMG), 37075 Göttingen, Germany; stephan.von.haehling@med.uni-goettingen.de

**Keywords:** hypertension, exercise intolerance, troponin, body mass compartments, personalization of body structure

## Abstract

The aim of this study was to identify factors influencing maximal oxygen uptake (VO_2max_) and early identification of the profile of hypertensive women in the perimenopausal period at risk of heart failure. This study included 185 female patients. Regression analyses determined predictors of the lowest VO_2max_ (quartile 1: VO_2max_ < 17 mL/kg/min). Females with the lowest oxygen consumption had a significantly higher level of high sensitive cardiac Troponin T (hs-cTnT) (*p* = 0.001), higher values of the left atrial (LA) volume, late diastolic mitral annulus velocity (A′), E/E′ (*p* = 0.0003, *p* = 0.02, *p* = 0.04; respectively), higher BMI and fat content (kg and %) (*p* < 0.0001), higher fat free mass (FFM) (kg) (*p* < 0.0001), total body water content (TBW) (*p* = 0.0002) as well as extracellular body water content (ECW) (*p* < 0.0001) and intracellular body water content (ICW) (*p* = 0.005), ECW/TBW × 100% (*p* < 0.0001) and metabolic age (*p* < 0.0001) and lower E′ (*p* = 0.001) compared to controls. In a multiple logistic regression model independently associated with VO_2max_ were: ECW/TBW × 100% (OR 4.45, 95% CI: 1.77–11.21; *p* = 0.002), BMI (OR 7.11, 95% CI: 2.01–25.11; *p* = 0.002) and hs-cTnT level (OR 2.69, 95% CI: 1.23–5.91; *p* = 0.013). High-sensitivity cardiac troponin may serve as an early biomarker of heart failure in hypertensive women. Hydration status should be considered in overall hypertensive women care. There is an importance of body mass compartments analysis in the early identification of hypertensive females at risk of heart failure. Optimization and personalization of body structure may be a preventive method for this disease. ClinicalTrials.gov Identifier: NCT04802369.

## 1. Introduction

Arterial hypertension is the most relevant factor of cardiovascular risk affecting circulatory diseases’ morbidity and mortality. It is estimated that 1.13 billion people in the world are diagnosed with hypertension [1,2]. An increasingly relevant problem in Europe, related to health and the economy, is heart failure (HF) [3]. The proportion of women is constantly growing in this group. Various studies have shown that progenitive factors, such as age at menopause, parity and polycystic ovarian syndrome (PCOS), may play a role in the development of HF [4,5]. Whether higher cardiovascular risk is a function of aging or a consequence of the menopause and its associated loss of endogenous estrogen has been debated in the literature for many years [6]. The loss of ovarian hormones around menopause has many adverse effects on coronary heart disease (CHD) risk factors. Hypertension is the most significant risk factor that affects women in the postmenopausal years. The onset of hypertension can cause a diversity of symptoms that are frequently attributed to menopause. Hypertension is also one of the most common conditions that can lead to HF. The Framingham study showed that hypertension was associated with 39% of HF cases in men and 59% in women. In both HF with reduced ejection fraction (HFrEF) and HF with preserved ejection fraction (HFpEF), women report a much lower quality of life as compared to men [7]. Many women over 40 years of age additionally to primary hypertension development report worsening exercise intolerance as the first symptom of HF. One of the most valuable tools to determine assessment of the functional capacity during exercise is cardiopulmonary exercise testing (CPET). The CPET provides diagnostic and prognostic data derived from the direct measurement of VO_2_ [8]. Therefore, we sought to determine clinical predictors of VO_2max_ decrease in middle-aged hypertensive women.

## 2. Materials and Methods

### 2.1. Basic Characteristics

In this study, 185 female patients with primary hypertension were enrolled. All subjects were hospitalized in the Department of Hypertension and Department of Cardiology and Congenital Heart Diseases of Adults between 2018 and 2020. A random sample in the electronic medical record was reviewed independently and in duplicate by two investigators (A.B.D. and K.G.) to validate the research strategy. All participants were consulted by a gynecologist at the Polish Mother’s Memorial Hospital Research Institute after 12 months from final menstrual period and were classified as patients during menopausal transition. All patients included in this analysis performed symptom-limited cardiopulmonary exercise testing (CPET) within one week. Physical activity level of accessed women was low. Their lifestyle was sedentary. Maximal oxygen uptake (VO_2max_) was reported as absolute maximal VO_2_, indexed to body weight and analyzed as quartiles, with quartile 1 representing the lowest VO_2max_. The patients were divided into a group that demonstrated maximal oxygen consumption measured during incremental exercise indexed per kilogram—VO_2max_ < 17 mL/kg/min (the lowest percentile) (54 women at the age 51 (±8) years old, median VO_2max_ 15 mL/kg/min) and a second group of women aged 55 (±8) years who presented VO_2max_ > 17 mL/min/kg (131 women, median 21 mL/kg/min). The study is in compliance with the Declaration of Helsinki and was approved by the Polish Mother’s Memorial Hospital Research Institute (PMMHRI-BCO.71/2020).

Exclusion criteria:

(a) uncontrolled hypertension;
(b) diagnosis of heart failure or typical symptomatic heart failure;
- left ventricular ejection fraction (LVEF) < 50%;
(c) documented: hyperandrogenism, hyperestrogenism, insulin resistance, premature ovarian failure, polycystic ovary syndrome;
(d) past myocardial infarction;
(e) diagnosis of cardiomyopathy (hypertrophic, dilated, restrictive, peripartum, arrhythmogenic);
(f) lysosomal storage disorders;
(g) stroke, transient ischemic attack, intracerebral hemorrhage in medical history;
(h) severe hyper- and hypothyroidism;
(i) pregnancy and lactation;
(j) chronic kidney disease (stage IV and V according to the National Kidney Foundation) and dialysis treatment;
(k) documented neoplastic process;
(l) the patient’s inability to cooperate and/or provide informed consent to participate in the research;
(m) alcohol and drug abuse;
(n) active autoimmune disease;
(o) treatment using immunosuppressants, cytostatic drugs, glucocorticosteroids, or antiretroviral drugs;
(p) a history of bone marrow transplant or other organ transplant, treatment with blood products within the last 6 months;
(q) active systemic infection;
(r) Hepatitis B virus (HBV), hepatitis C virus (HCV) or human immunodeficiency virus (HIV) carrier or positive for hepatitis B surface antigen (HBsAg) or antibodies to HCV;
(s) surgery or serious injury within the last month;
(t) patients who did not express their informed consent to participate in the study.

### 2.2. Echocardiography

The patients underwent echocardiography using the Vivid E95 system (GE Healthcare, Chicago, IL, USA). Numerical measures were conducted according to current guidelines [9]. Left ventricular (LV) volume and ejection fraction (EF) were derived according to the modified biplane Simpson’s rule. Left atrial (LA) volume was obtained using the modified biplane Simpson’s method and indexed to body surface area (LA volume index—LAVi) [10]. Residual echocardiographic parameters analyzed were the ratio of early transmitral peak velocity to early diastolic peak annular velocity (E/E′). Early diastolic (E′) and late diastolic (A′) mitral annular myocardial velocity of the left ventricle were recorded using pulsed-wave Doppler from the apical 4-chamber view [11]. The right ventricular (RV) measure was tricuspid annular plane systolic excursion (TAPSE).

### 2.3. Laboratory Tests

Diagnostic blood samples were collected from each patient. The samples were obtained by needle puncture and withdrawn by suction through the needle into a vacuum blood collection system. Laboratory tests were performed in the hospital laboratory. We measured liver function (alanine aminotransferase (ALT) and aspartate transaminase (ASP)); renal function (creatinine, glomerular filtration rate (GFR) estimate by Modification of Diet in Renal Disease (MDRD)) parameters, inflammatory cytokine (high-sensitivity C-reactive protein (CRP)), glucose level, lipoprotein profile: low-density lipoprotein (LDL), high-density lipoprotein (HDL) and triglycerides (TG). Additionally, the amount of hemoglobin was measured, and the analysis of N-terminal pro B-type natriuretic peptide (NT-proBNP) and high-sensitivity cardiac troponin T (hs-cTnT) was conducted. Only high-sensitivity troponin values were used for further analysis. Biochemical tests were performed using a biochemical analyzer (AU 640 Olympus, Tokyo, Japan). The determination of high-sensitivity CRP was performed in plasma using a certified latex particle-enhanced immunologic turbidimetric assay. Plasma-glucose concentration was obtained using the hexokinase method (Olympus OSR61221, OSR6221 Tokyo, Japan). Creatinine concentrations in plasma samples were measured with a rate-blanked and compensated picric acid colorimetric assay (Olympus OSR6178, Tokyo, Japan). A spectrophotometric method for quantitative determination of hemoglobin was used. Plasma and serum were separated by centrifugation and serum hs-cTnT and NT-proBNP were measured in plasma using enzyme-linked immunosorbent assay (ELISA) tests (Roche Diagnostics, Warsaw, Poland).

### 2.4. Spiroergometry

Symptom-limited cardiopulmonary exercise testing (CPET) was performed on an electromagnetically braked upright cycle ergometer Bike M (CORTEX Biophysik GmbH, Leipzig, Germany) with a metabolic gas analyzer METALYZER 3B (CORTEX Biophysik GmbH, Leipzig, Germany) using the MetaSoft Studio application software (CORTEX Biophysik GmbH, Leipzig, Germany) [12]. Exercise testing on a bicycle ergometer was preceded by spirometry. Forced vital capacity (FVC) and forced expiratory volume in one second (FEV1) were estimated. We also recorded FEV1/FVC ratio (Tiffeneau index). CPET on a bicycle ergometer was conducted with additional continuous 12-lead electrocardiogram (ECG), heart rate (HR), peripheral oxygen saturation (SpO_2_) and non-invasive blood pressure (NIBP) monitoring. Exercise test had three stages: a 1 min rest phase where the patient sat on the ergometer; cycling at a constant cadence, despite changing resistance and a further 5 min of recorded rest. Initially the workload was 20 watts (W) and increased by 25 W every 3 min. One of the most important measurements is VO_2max_ (the maximal rate of muscle oxidative metabolism). If a plateau is not obtained during CPET, the highest VO_2_ attained is the VO_2_ peak and can be used as a substitute for VO_2max_ [13]. We also assessed other valuable CPET parameters. These derived measurements included ventilatory exchange (VE), oxygen uptake (VO_2_), CO_2_ expenditure (VCO_2_), respiratory exchange ratio (RER), anaerobic threshold (AT), oxygen uptake at anaerobic threshold (VO_2_ AT), and the minute ventilation/carbon dioxide production slope (VE/VCO_2_ slope).

### 2.5. SphygmoCor

The SphygmoCor 9.0 tonometer (AtCor Medical, Sydney, Australia) is a tool for non-invasive assessment for central arterial pressure waveform analysis [14]. We obtained the aortic systolic pressure (SP aortic), aortic diastolic pressure (DP aortic) and aortic pulse pressure (PP aortic) by using this method. Additionally, the parameters of arterial stiffness were measured: augmentation pressure (AP), augmentation index (AIx) and pulse wave velocity (PWV). AP is the difference between the first and second systolic peaks on the central pressure waveform [15]. AIx was calculated by AP as a percentage of the entire pressure waveform height. PWV is the path length divided by transit time.

### 2.6. Body Mass Analysis

Non-invasive body mass analysis was performed using the Segmental Body Composition Analyzer (Tanita Pro, Tokyo, Japan). After the gender, age and height information had been entered into the device, patients were asked to stand barefoot in a stable position. The implement provided body mass analysis for legs, arms and whole body—using an algorithm incorporating impedance, age and height, to assess total and regional fat mass (FM) and fat-free mass (FFM) [16]. We also measured the following parameters: total body water (TBW), and extracellular (ECW) and intracellular (ICW) water. Additionally, extracellular water ratio normalized for total body water (ECW/TBW × 100%) was calculated [17]. ECW/TBW × 100% is a significant indicator of body water balance.

### 2.7. Statistical Analysis

The STATISTICA 13.1 software package (StatSoft, Kraków, Poland) was used for analysis. The Shapiro–Wilk test assessed the normality of distribution. The two-tailed Student’s *t*-test for normal distributed data and Mann–Whitney U test for non-normally distributed variables were used. The receiver-operating characteristic curve (ROC) for significant continuous data in univariate analyses was prepared and the Youden index was used to transform these data from continuous into categorical ones. These categorical data were tested by backward stepwise multivariate logistic regression. All results were considered significant at *p* < 0.05.

## 3. Results

### 3.1. Evaluation of Basic Characteristics

Demographics, clinical characteristics, biochemical parameters and biomarkers according to VO2 quartiles (the lowest characteristic compared with better oxygen consumption) are presented in Table 1. Average age was lower in the group with the lowest oxygen consumption (51 (±8) vs. 55 (±8), *p* = 0.005). Body mass and body mass index (BMI) were significantly higher in women with the lowest exercise capacity (median 81.45 vs. 68.8 kg, *p* < 0.0001 and median 30.34 vs. 25.85 kg/m^2^, *p* < 0.0001, respectively). Women with the lowest oxygen consumption had a significantly higher level of hs-cTnT (median 4.5 vs. 3.4 pg/mL, *p* = 0.001) although there was no difference in NT-proBNP level. The differences in other characteristics were not statistically significant. Data are presented in Table 1.

### 3.2. Evaluation of Echocardiographic and Hemodynamic Parameters

LA volume, A′ and E/E′ were significantly higher (median 62.5 vs. 52 mL, *p* = 0.0003; median 11 vs. 10 cm/s, *p* = 0.02; median 8.2 vs. 7.68 cm/s, *p* = 0.04; respectively) and E′ was lower (median 8.5 vs. 10 cm/s, *p* = 0.001) in women with VO_2max_ < 17 mL/kg/min compared to controls. There were no statistically significant differences regarding LVEF, LAVi, TAPSE, (*p* = 0.7; *p* = 0.07; *p* = 0.96; respectively). The hemodynamic parameters assessed non-invasively using the SphygmoCor system had no effect on oxygen consumption in the middle-age female population. Results are presented in Table 2.

### 3.3. Evaluation of Cardiocirculatory, Pulmonary and Metabolic Response to Exercise in CPET

Patients with the lowest oxygen consumption had lower FEV1 and FVC (median 2.63 vs. 2.71 l, *p* = 0.01; median 3.15 vs. 3.34, *p* = 0.001; respectively) and a higher Tiffeneau index (FEV1/FVC%) (median 108 vs. 105%, *p* = 0.02) compared to patients with better exercise tolerance. Exercise time was significantly longer (9.05 (±2.07) vs. 7.28 (±1.93) min, *p* < 0.0001) and HRmax was higher (150.06 (±16.05) vs. 127.17 (±19.89), *p* < 0.0001) in patients in the lowest percentile of VO_2max_. Metabolic gas exchange measurements showed lower values of VO_2_AT, peak VO_2_, highest VO_2max_, VE/VCO_2_ slope (median 10 vs. 13 mL/min/kg, *p* < 0.0001; median 1.24 vs. 1.45 l, *p* < 0.0001; median 15 vs. 21 mL/min/kg, *p* < 0.0001; 27.98 (±3.71) vs. 29.35 (±4.55), *p* = 0.04; respectively) and slightly lower RER (median 1.07 vs. 1.14, *p* < 0.0001) (Table 3).

### 3.4. Evaluation of Body Mass Analysis

Female patients with the lowest exercise tolerance in CPET had significantly higher BIA parameters: levels of fat as a percentage and in kilograms (median 38.3 vs. 33.75%, *p* < 0.0001; median 30.4 vs. 23.8 kg, *p* < 0.0001; respectively), FFM (median 53.8 vs. 44.8 kg, *p* < 0.0001); TBW (median 36.25 vs. 32.4 kg, *p* = 0.0002); ECW (median 16.7 vs. 14.4, *p* < 0.0001); ICW (median 19.3 vs. 18, *p* = 0.005); ECW/TBW × 100% (median 46.5 vs. 45, *p* < 0.0001); metabolic age (median 61 vs. 47, *p* < 0.0001) compared to counterparts. TBW percentage was lower (median 43.7 vs. 47.1, *p* < 0.0001) in patients with VO_2max_ < 17 mL/kg/min than in controls. Results are presented in Table 4.

### 3.5. Multivariate Analysis

**Figure 1:**
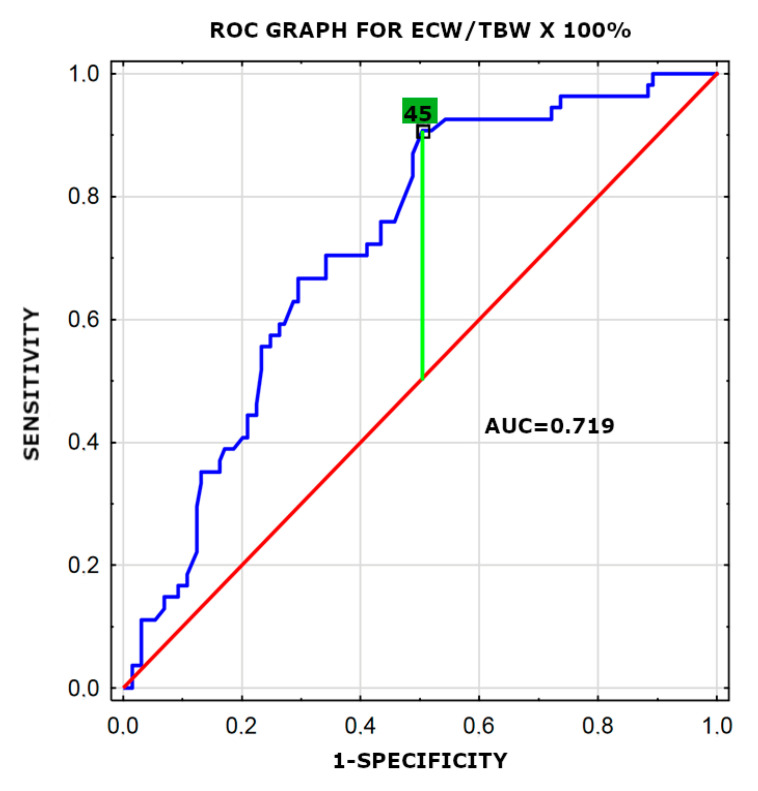
Receiver-operating characteristic curve (ROC) for the ECW/TBW × 100% variable revealing its diagnostic potential. AUC -area under the ROC curve.

**Figure 2:**
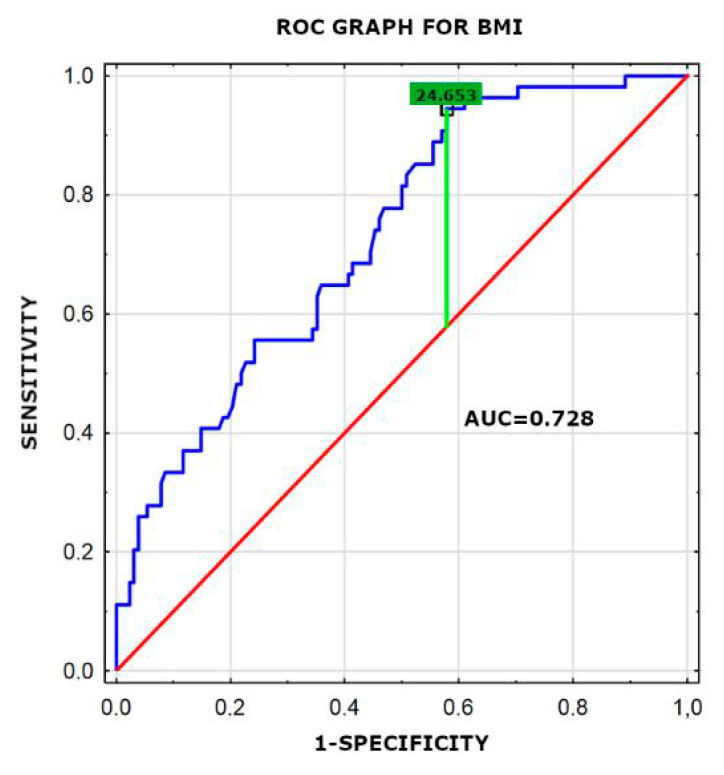
Receiver-operating characteristic curve (ROC) for the BMI variable revealing its diagnostic potential. AUC -area under the ROC curve.

Parameters with a *p* value < 0.05 in the univariate analysis were entered into the multivariate analysis using the logistic regression analysis. In a multiple logistic regression model, three factors were found to be significantly associated with VO_2max_: ECW/TBW × 100% (Figure 1) (OR 4.45, 95% CI: 1.77–11.21; *p* = 0.002), BMI (Figure 2) (OR 7.11, 95% CI: 2.01–25.11; *p* = 0.002) and hs-cTnT level (OR 2.69, 95% CI: 1.23–5.91; *p* = 0.013). Results are presented in Table 5. The value of ECW/TBW × 100% higher than 45, BMI > 24.65 and hs-cTnT level higher than 3.3 pg/mL were associated with VO_2max_ < 17 mL/kg/min.

## 4. Discussion

The current study shows that selected parameters of diastolic left ventricular dysfunction and increased high-sensitivity troponin but not NT-proBNP levels and parameters of arterial stiffness, or central and peripheral blood pressures are connected with the lowest oxygen uptake assessed in CPET in hypertensive women. Pulmonary function measured in spirometry was worse and the assessment of the exercise response parameters in CPET were lower in patients with VO_2max_ < 17 mL/kg/min than in the group with better exercise capacity. Women in the lowest percentile of VO_2max_ also had significantly higher BMI, fat content (kg and %), higher FFM (kg), higher TBW as well as ECW and ICW, ECW/TBW × 100% and metabolic age compared to counterparts. TBW percentage, however, was lower in patients with VO_2max_ < 17 mL/kg/min than in controls. In a multiple logistic regression model, the following factors were independently associated with VO_2max_: ECW/TBW × 100% (higher than 45), BMI (>24.65) and hs-cTnT level (>3.3 pg/mL).

Exercise capacity has become a well-established independent predictor for cardiovascular disease [18]. VO_2,_ expressed as VO_2_ per kilogram, is used to investigate the performance of the respiratory-cardiovascular system. This parameter offers the best risk stratification across the heart failure cohort including women and the obese [19]. Hulens et al. assessed the differences in submaximal and maximal exercise capacity parameters between 81 lean and 225 obese women. At a submaximal intensity load of 70 W, oxygen uptake (VO_2_) was larger in the obese women and was already 78% of their peak VO_2_, whereas in the non-obese it was only 69% (*p* = 0.0001). Slim females recovered better. After 2 min they were already at 35% of the peak VO_2_, while in the obese females it was 47% (*p* = 0.0001). These results confirm that exercise capacity is decreased in obesity and are in accordance with our results, where women with the lowest exercise capacity had higher BMI and fat mass [20]. Babb et al. also supported our results in the study which investigated the effect of moderate obesity on ventilatory responses to graded exercise and compared the ventilatory responses of ten moderately obese (35 +/− 5% body fat) and nine slim women (22 +/− 2% body fat) during an incremental treadmill walking test. At 10.0% and 12.5% grade, respiratory oxygen uptake (VO_2_) was lower and ventilatory equivalents for oxygen were larger in the obese women. The difference in VE/VO_2_ suggested a lower ventilatory threshold for the obese women [21].

Cardiac troponin T (cTnT) and troponin I (cTnI) are cardiac proteins that control the interaction between actin and myosin [22]. The troponin complex is located on the myofibrillar thin (actin) filament of striated (skeletal and cardiac) muscle. The levels of cTnI and TnT were evaluated using monoclonal antibodies in immunometric assay formats resulting in clinical assays specific to myocardial injury [23]. In the study of Ebong et al. the authors investigated the effects of early menopause (occurrence before 45 years of age) and hs-cTnT elevation (≥ 14 ng/L) on heart failure (HF) incidence in 2276 postmenopausal women aged 67–90 years. The hs-cTnT elevation was related to higher HF incidence with or without early menopause (3.03 (95% CI, 1.59–5.77)) and (3.29 (95% CI, 2.08–5.21)), respectively, but this relationship was partly explained by HF risk factors [24].

Extracellular fluid status was defined as extracellular water to total body water ratio (ECW/TBW) measured using bioelectrical impedance analysis. The measurement of ECW/TBW ratio is a simple and fast indicator of hydration status. The ECW/TBW ratio is used as a marker of the extracellular volume status. The study of Waki et al. indicated that fluid volumes are increased in obese women, and the expansion is relatively greater for the extracellular compartment [25]. In another study of 95 participants with end-stage chronic kidney disease on dialysis therapy, ECW/TBW ratio presented the strongest correlation with decreased exercise tolerance (R = −0.63; *p* < 0.001) [26].

In our study, a value of ECW/TBW × 100% higher than 45 and BMI > 24.65 were independently associated with the lowest exercise tolerance in hypertensive women. There is a paucity of research assessing the relationship between fluid distribution, body mass and exercise tolerance in hypertensive women aged over 40 in stage A heart failure. Our findings provide new evidence in this field and indicate that therapeutic strategies targeting fluid status and body mass compartments may increase the exercise capacity of middle-age females with hypertension.

There are several limitations of the present study, which included a small study population (185 women in the perimenopausal period) without healthy controls. The patient population was limited to those hospitalized due to HA in our department, which could have caused referral bias because patients referred for hospitalization were not representative of the general HA population. Because the disease severity in our patients was mild or moderate, the results should be carefully interpreted when applied to different populations. In addition, the study design was limited regarding the evaluation of the effect of medications. Moreover, an echocardiogram was performed only at rest. Finally, the present study only included stable patients who were able to undergo CPET. Hence, the present results need to be interpreted with caution and should be reproduced in a larger HA population, preferably in a prospective controlled clinical trial.

Despite these limitations, this is to our best knowledge the first study of hypertensive women in the perimenopausal period to evaluate the risk of heart failure, assessing the impact of high-sensitivity cardiac troponin level on the development of heart failure in hypertensive female patients in perimenopausal age. We demonstrated that overhydration also played an important role in HA patients and the risk of heart failure. Hydration status should be considered in overall patient care.

## 5. Conclusions

High-sensitivity cardiac troponin may serve as an early biomarker of heart failure in hypertensive women. Hydration status should be considered in the overall hypertensive care of women. Body mass compartments analysis in early identification of hypertensive females at risk of heart failure. Optimization and personalization of body structure may be a preventive method for this disease.

## Figures and Tables

**Table 1:** Evaluation of basic characteristics.

Parameter	Group in the Lowest Percentile of VO_2max_ (<17 mL/kg/min) *n* = 54	Group in Higher Percentiles of VO_2max_ *n* = 131	*p*
Age	51.28 (±8.13)	54.94 (±7.54)	0.005
Height (cm)	(160–167), 164 *	(160–167), 163 *	0.28
Body mass (kg)	(71.8–93.5), 81.45 *	(62.1–80.2), 68.8 *	<0.0001
BMI (kg/m^2^)	(26.80–34.76), 30.34 *	(22.93–29.71), 25.85 *	<0.0001
Glucose (mg/dL)	(87–99), 93 *	(87–97), 92 *	0.54
HDL cholesterol (mg/dL)	(36–56), 47 *	(42–61.5), 51 *	<0.05
LDL cholesterol (mg/dL)	113.51 (±31.23)	118.58 (±36.05)	0.42
Triglycerides (mg/dL)	(80–183), 124 *	(82–153), 114 *	0.42
Hemoglobin (g/dL)	(12.5–13.9), 13.45 *	(12.5–14.2), 13.5 *	0.64
GFR (mL/min/1.73 m^3^)	(75.8–96.1), 85.25 *	(76.5–100.3), 86.1 *	0.6
ALT (U/L)	(14–27), 20 *	(14–27), 19 *	0.98
AST (U/L)	(20–26), 23 *	(19–26), 22 *	0.46
hs-CRP (mg/L)	(0.5–0.76), 0.5 *	(0.5–0.56), 0.5 *	0.1
hs-cTnT (pg/mL)	(3.4–5.9), 4.5 *	(3–4.7), 3.4 *	0.001
NT-proBNP (pg/mL)	(37–140), 71 *	(40–97), 64 *	0.32

*—median; Values with non-normal distribution are expressed as median (range) values. Values with normal; distributions are expressed as mean ± standard deviation (SD). BMI—body mass index; ALT—alanine aminotransferase; AST—aspartate aminotransferase; hs-CRP—high-sensitivity c-reactive protein; GFR—glomerular filtration rate; HDL—high-density lipoprotein; LDL—low-density lipoprotein; hs-cTnT—high-sensitivity cardiac troponin; NT-proBNP—N-terminal prohormone of brain natriuretic peptide.

**Table 2:** Evaluation of selected echocardiographic and hemodynamic parameters among the groups.

Parameter	Group in the Lowest Percentile of VO_2max_ (<17 mL/kg/min) *n* = 54	Group in Higher Percentiles of VO_2max_ *n* = 131	*p*
EF (%)	(61–65), 64 *	(61–67), 64 *	0.7
LA volume (mL)	(55–71.5), 62.5 *	(45.5–65.5), 52 *	0.0003
LAVi (mL/m^2^)	(28.35–38.9), 34.03 *	(27.24–35.84), 30.12 *	0.07
E′ (cm/s)	(8–10.5), 8.5 *	(8.5–12), 10 *	0.001
A′ (cm/s)	(10–12.5), 11 *	(9–12), 10 *	0.02
E/E′ (cm/s)	(7.2–10), 8.2 *	(6.56–8.7), 7.68 *	0.04
TAPSE (mm)	(22–26), 23 *	(21–27), 23 *	0.96
PWV (m/s)	(6.9–8.8), 7.65 *	(6.8–8.5), 7.4 *	0.36
Aortic SP (mmHg)	(112–130), 119 *	(109–129), 118 *	0.36
Aortic PP (mmHg)	(41–80), 68.5 *	(41–80), 63 *	0.64
AP (mmHg)	(9–17), 13 *	(10–15), 12 *	0.5
Alx (%)	(23–42), 31.5 *	(27–37), 32 *	0.91
Alx@HR75 (%)	(22–36), 27.5 *	(23–35), 32 *	0.74

*—median; Values with non-normal distribution are expressed as median (range) values. Values with normal distributions are expressed as mean ± standard deviation (SD). aortic SP—aortic systolic pressure; aortic PP—aortic pulse pressure; AP—augmentation pressure; AIx—augmentation index; AIx@HR75—adjusted augmentation index at heart rate 75/min; A′—late diastolic mitral annulus velocity; E/E′—ratio of peak velocity of early diastolic transmitral flow to peak velocity of early diastolic mitral annular motion as determined by pulsed wave Doppler; E′—early diastolic mitral annular velocity; LA—left atrium; LAVi—left atrial volume index; LV—left ventricle; EF—left ventricular ejection fraction; PWV—pulse wave velocity; TAPSE—tricuspid annular plane systolic excursion.

**Table 3:** Evaluation of spiroergometric parameters.

Parameter	Group in the Lowest Percentile of VO_2max_ (<17 mL/kg/min) *n* = 54	Group in Higher Percentiles of VO_2max_ *n* = 131	*p*
Exercise time (min)	9.05 (±2.07)	7.28 (±1.93)	<0.0001
HR max	150.06 (±16.05)	127.17 (±19.89)	<0.0001
Peripheral SBP max (mmHg)	(160–190), 180 *	(160–190), 170 *	0.88
Peripheral DBP max (mmHg)	(80–90), 80 *	(80–90), 80 *	0.44
FEV1 (L)	(2.19–2.87), 2.63 *	(2.48–2.98), 2.71 *	0.01
FVC (L)	(2.48–3.34), 3.15 *	(2.97–3.75), 3.34 *	0.001
FVC%	(95–115), 105 *	(101.5–123.5), 111 *	0.001
FEV1/FVC	(79–88), 85 *	(78–87), 83 *	0.13
FEV1/FVC%	(100–113), 108 *	(98–109), 105 *	0.02
FEF 25–75	(1.65–3.13), 2.43 *	(1.88–3.19), 2.59 *	0.13
RER	(1.01–1.12), 1.07 *	(1.09–1.18), 1.14 *	<0.0001
VO_2max_ (mL/min/kg)	(14–16), 15 *	(18–23), 21 *	<0.0001
VO_2_AT (mL/min/kg)	(9–11), 10 *	(12–15), 13 *	<0.0001
Peak VO_2max_ (L/min)	(1.09–1.4), 1.24 *	(1.29–1.64), 1.45 *	<0.0001
VE/VCO_2_ slope	27.98 (±3.71)	29.35 (±4.55)	0.04

*—median; Values with non-normal distribution are expressed as median (range) values. Values with normal distributions are expressed as mean ± standard deviation (SD). DBP—diastolic blood pressure; SBP—systolic blood pressure; FEV1—forced expiratory volume in one second; FVC—forced vital capacity; FEV1/FVC—ratio of forced expiratory volume in one second to forced vital capacity; FEF 25–75%—forced expiratory flow over the middle one half of the FVC; RER—respiratory exchange ratio; VO_2max_—the maximum amount of oxygen the body can utilize during a specified period of usually intense exercise; VO_2_AT—oxygen uptake at anaerobic threshold per kilogram; peak VO_2_—highest respiratory oxygen uptake (VO_2_) achieved by the subject during the maximal exercise; VE/VCO_2_ slope—the minute ventilation/carbon dioxide production slope.

**Table 4:** Evaluation of body composition parameters.

Parameter	Group in the Lowest Percentile of VO_2max_ (<17 mL/kg/min) *n* = 54	Group in Higher Percentiles of VO_2max_ *n* = 131	*p*
Fat (%)	(35.8–40.8), 38.3 *	(29.45–38.1), 33.75 *	<0.0001
Fat (kg)	(27.3–37.7), 30.4 *	(17.75–29.5), 23.8 *	<0.0001
FFM (kg)	(50.8–56.8), 53.8 *	(42.1–47.2), 44.8 *	<0.0001
TBW (kg)	(32.1–39.5), 36.25 *	(30.5–35.7), 32.4 *	0.0002
TBW (%)	(42–45.3), 43.7 *	(43.9–50.3), 47.1 *	<0.0001
ECW (kg)	(14.8–18.3), 16.7 *	(13.6–16.2), 14.4 *	<0.0001
ICW (kg)	(17.5–21), 19.3 *	(16.5–19.5), 18 *	0.005
ECW/TBW × 100%	(45.45–47.28), 46.5 *	(43.75–46.28), 45 *	<0.0001
Metabolic age	(54–67), 61 *	(40–59), 47 *	<0.0001

*—median; Values with non-normal distribution are expressed as median (range) values. Values with normal distributions are expressed as mean ± standard deviation (SD). ECW—extracellular water; FFM—fat-free body mass; ICW—intracellular water, ECW/TBW%—ratio of extracellular water to total body water; TBW—total body water.

**Table 5:** Multivariate analysis—stepwise logistic regression.

Variable	OR	95% CI for OR		*p*
		Lower Limit	Upper Limit	
ECW/TBW × 100%	4.45	1.77	11.21	0.002
BMI	7.11	2.01	25.11	0.002
hs-cTnT	2.69	1.23	5.91	0.013

ECW/TBW—extracellular water to total body water ratio; BMI—body mass index; hs-cTnT—high-sensitivity cardiac troponin T; OR—odds ratio; CI—confidence interval.

## Data Availability

Individual participant data that underlie the results reported in this article after deidentification (text, tables, figures and appendices) as well as study protocol will be available for researchers who provide a methodologically sound proposal. Proposals may be submitted after 9 months and up to 36 months following article publication.
